# Intra- and Interobserver Variability in Ultrasound Measurement of Testicular Volumes in Pubertal Boys

**DOI:** 10.3390/children11060741

**Published:** 2024-06-17

**Authors:** Frank-Mattias Schäfer, Daniel Bürgener, Maximilian Stehr, Oliver Rompel

**Affiliations:** 1 Cnopfsche Kinderklinik, Department of Pediatric Surgery and Pediatric Urology, 90419 Nürnberg, Germany; daniel.buergener@diakoneo.de (D.B.); maximilian.stehr@diakoneo.de (M.S.); 2 Clinic of Urology and Pediatric Urology, University Hospital Erlangen, Friedrich-Alexander-Universität (FAU) Erlangen-Nürnberg, 91054 Erlangen, Germany; 3 Institute of Radiology, University Hospital Erlangen, Friedrich-Alexander-Universität (FAU) Erlangen-Nürnberg, 91054 Erlangen, Germany; oliver.rompel@uk-erlangen.de

**Keywords:** testicular size, testicular ultrasound, varicocele, intraobserver variability, interobserver variability

## Abstract

Accurate measurement of testicular volume (TV) in boys is an important tool in clinical practice, e.g., in varicocele treatment. This study aims to assess the degree of intra- and interobserver variability of testicular volume measurements. In a prospective study, boys between 11 and 17 years of age without testicular pathology were enrolled. Testicular ultrasound was performed by three investigators (A: pediatric radiologist; B: pediatric surgery/urology resident; C: pediatric urologist). Intraobserver variability was calculated in investigators B and C and interobserver variability between all three investigators. A total of 30 boys were enrolled. Mean intraobserver variability in both observers was +0.3% with a range of −39.6 to 51.5%. The proportion of measurements with a difference >20% was 18.6%. The mean interobserver variability was −1.0% (range: −74.1% to 62.8%). The overall proportion of measurements with a difference >20% was 35%. A lower testicular size of < 4 mL showed a significantly higher rate of >20% difference in both the intraobserver group (31.1% vs. 14.4%; *p* = 0.035) and the interobserver group (63.2% vs. 26.2%; *p* = 0.000031). Furthermore, the rate of >20% difference was significantly lower in obese compared to non-obese patients in both the intraobserver (2.8% vs. 22.4%; *p* = 0.0084) and the interobserver group (24% vs. 40.8%, *p* = 0.0427). Both intraobserver and interobserver variability in ultrasound-based TV measurements in pubertal boys contain a relevant degree of uncertainty that renders them unsuitable for individualized follow-up care. At the cohort level, however, mean differences in ultrasound-based TV measurements are low enough to make ultrasound comparisons reasonable.

## 1. Introduction

The accurate measurement of testicular volume (TV) in boys is an important tool in both clinical practice and longitudinal studies of puberty onset. For example, EAU guidelines note that in adolescents, a testis that is smaller by >2 mL or 20% compared to the other testis is considered to be hypotrophic, and varicocele correction is recommended in patients with a (persistent) small testis [1]. As testicular growth may be physiologically asynchronous in over 50% of patients [2], it is necessary to obtain serial measurements before decision making [3]. The issue is further complicated by the fact that testicular growth undergoes a rapid increase during puberty, which leads to vast differences in testicular volume [4]. This makes the indication for varicocele repair based on absolute volume differences (in mL), as mentioned above, appear doubtful or potentially leads to the 20% limit being exceeded more quickly in smaller testes given the same level of measurement inaccuracy [5]. 

While testicular volume estimation with a Prader orchidometer can still be useful in some clinical situations, several studies have shown that it is too insensitive to measure volume differentials to determine growth impairment [6] and that ultrasound techniques are more accurate than an orchidometer when compared to water displacement methods [7,8]. This holds true also in patients with varicocele where an orchidometer compared to ultrasound has a low sensitivity of 33% at the 20% differential volume level, potentially missing two-thirds of affected boys [9].

On the other hand, there still exists a profound controversy of the reliability of ultrasound testicular measurements, especially as serial measurements are required in many clinical scenarios. In one recent study, no significant mean intra- and interobserver difference was found [10]. The authors observed a very limited intraobserver error and supported the use of ultrasound measurements in longitudinal follow-up. In contrast to this, Welliver et al. found a considerable intra- and interobserver variability in boys aged 11 to 19 years, with a rate of >20% volume difference in over 25% of the patients [11]. They conclude that caution is needed when using ultrasound measurements as a basis for clinical decisions and scientific studies [11]. On the other hand, in adults Pedersen et al. demonstrated a low variability in estimating testicular volume, with an excellent interobserver agreement. They concluded that ultrasound provides a highly reproducible tool to determine testicular volume [12].

This uncertainty illustrates the need for further investigations, especially as there is no gold standard available to which ultrasound measurements can be compared and as there are potentially other influencing factors which include BMI or baseline testicular volume as a marker of pubertal testicular development. This study aims to provide thorough data on intra- and interobserver variability in a larger dataset with repeated measurements, focusing on identifying the conditions under which reliable testicular volume measurement is possible.

## 2. Materials and Methods

After procurement of Institutional Ethics Committee approval (ref. no.: 22-9-B), boys hospitalized at our children’s hospital for other reasons than testicular pathology (such as epididymitis, testicular torsion, tumor, scrotal trauma, or endocrinological diseases which affect testicular development) and without previous history of testicular pathology between 11 and 17 years of age were prospectively enrolled into the study. Informed consent was obtained both from the boys as well as both parents. Patients with incidental findings of testicular or scrotal pathology were excluded from further analysis. 

All patients were examined by three investigators: (A) a pediatric radiologist (>20 years of experience), (B) a pediatric urology consultant (>15 years of experience), and (C) a pediatric surgery resident (5 years of experience). Intraobserver variability was calculated in investigators B and C, who performed two measurements each (B1/B2 and C1/C2). Interobserver variability was calculated using all three investigators’ measurements (A-B1, A-C1, and B1-C1).

### 2.1. Examination Conditions

**Figure 1:**
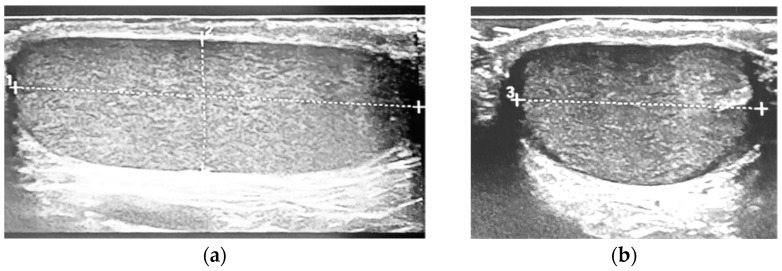
Example of the standardized ultrasound technique used: (**a**) length (dotted line 1) and depth (dotted line 2) measured on the sagittal view; (**b**) the probe is then placed per-pendicular and the widthwidth (dotted line 3) is obtained at the broadest point.

The probands were in prone position with slightly spread legs. No scrotal pad was used to support the testicles. Measurements of both testicles were performed using a copious amount of warmed coupling gel in two standardized directions to allow for optimal accuracy: A longitudinal measurement with slight angulations in both directions was carried out first to determine the largest longitudinal diameter (L). In this setting, the height (H) of the testicle was also measured at its widest point. The second measurement was then carried out in an axis perpendicular to the first measurement to determine the width (W) of the testicle (Figure 1).

All measurements were taken on the same device (GE Logic S8 XDclear, GE Healthcare, Chicago, IL, USA) with a ML6-15-D Matrix linear probe at 10 MHz using standardized scrotal imaging settings. For measurement of testicular volume, Lambert’s formula (L × H × W × 0.71) was used, which has been shown to be the most accurate formula available [8,13]. The examinations were performed within three days, and the investigators were blinded to the other’s measurements as well as their own previous measurements. Additional parameters obtained were age, BMI, and Tanner stage of the genitalia. Age-correlated BMI percentile was calculated according to the German population and age-related distribution [14]. Overweight was defined as a BMI percentile >90.

### 2.2. Statistical Analysis

Data processing and calculation was performed using GraphPad Prism^®^ 10.2.2 (GraphPad Software Inc., Boston, MA, USA) and Microsoft Excel^®^ 365 (Microsoft Corporation, Washington, DC, USA). Paired *t*-test, chi-square test, fixed effects models, one-sided ANOVA (fixed effects model), linear regression analysis, Pearson’s r correlation, and Bland–Altman plots were used to analyze the data. Bland–Altman analysis is especially suited for visualizing variability as it does not require a gold standard (such as TV water displacement in this case) [15]. In Bland–Altman scatter plots, the differences between two measurements are plotted against their averages to visualize the degree of agreement between the two observers and to identify any substantial systemic bias. *p* values < 0.05 were considered statistically significant. A sample size estimate showed that with an effect size of d = 0.4 and a power of 0.8, a total of 52 testicular units are required to achieve a statistically significant result with a two-sided paired *t*-test (α = 0.05).

## 3. Results

A total of 35 boys were recruited for the study. Four patients were excluded from the analysis because of incidental findings of left-sided varicocele (2x) and epididymal cysts (2x). One patient retracted his consent after the first ultrasound and was therefore also removed from the study. Thus, a total of 30 patients (60 testicular units) were included for further analysis. The underlying cause for hospital treatment in the patients was mainly pediatric orthopedic/trauma (20 patients), general pediatric surgery (5), and general pediatrics (2). Only three patients were from the pediatric urology department. 

In six patients, one measurement each was missing because of non-availability of one of the observers, resulting in a total of 288 TV difference comparisons. 

Basic demographic data of the patients are summarized in Table 1. Mean testicular volume was 9.2 mL (range: 0.5–27.5 mL) over all measurements, thus indicating the vast range of testicular volume during puberty. Mean volumes of left vs. right testes did not differ significantly (left TV: 9.26 mL, right TV: 9.22 mL).

BMI was not available in two patients due to missing values for weight or body length. The mean BMI percentile was 60.9, indicating a larger proportion of overweight patients, probably due to the disproportionate share of orthopedic patients who potentially are less physically active due to an impairment of their musculoskeletal system. Indeed, 30% of the patients (9/30) had a BMI percentile > 90, which is considerably higher than the expected percentage of approx. 18% according to current population-wide data [14]. 

**Figure 2:**
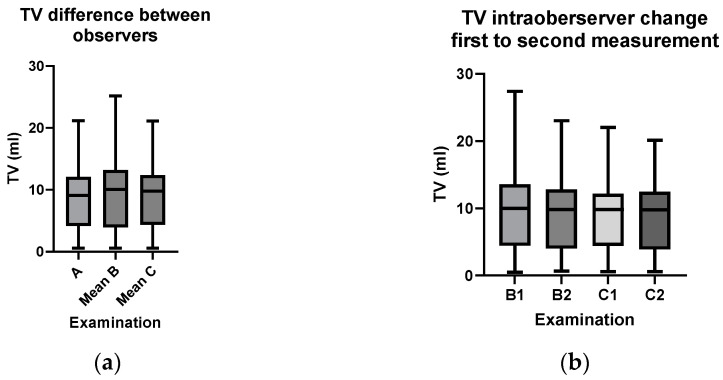
(**a**) Comparison of TV between the three observers. Because observers B and C had two measurements each, their mean was used. Mean TV A: 8.92 ± 0.77 mL, B (mean): 9.51 mL ± 0.80 mL, C (mean): 9.04 ± 0.70 mL. The results were significant (*p* = 0.017, fixed effects model), showing slightly different TV measurements in different observers. (**b**) Change in TV in intraobserver group: comparing measurement 1 to measurement 2 showed a decrease in range in measurement 2 in both observers, indicating a decrease in spread, but no significant change in TV (B1: 9.58 ± 0.80 mL, B2: 9.56 mL ± 0.80 mL, C1: 9.21 ± 0.73 mL, C2: 8.89 mL ± 0.68 mL (B1→B2: *p* = 0.58, C1→C2: *p* = 0.10; paired *t*-test).

Mean TV did differ slightly but significantly between the different observers (Figure 2a), but not according to the length of clinical practice. When comparing the change in TV in the intraobserver group over time, i.e., from measurement 1 to measurement 2, in the second measurements a reduction in range but no significant difference in TV between the examinations was noted (Figure 2b). In conclusion, experience (either in years of clinical practice or having seen the same patient twice) did not make any difference in TV measurement.

### 3.1. Intraobserver Variability

**Figure 3:**
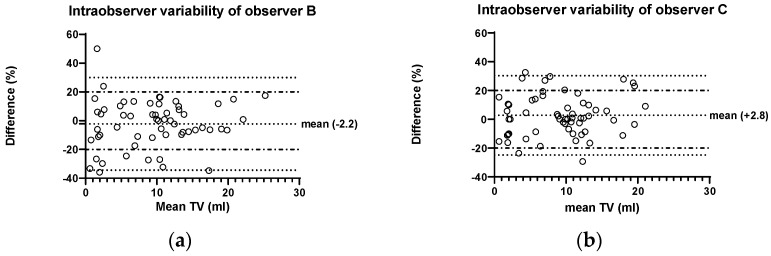
Bland–Altman diagram of intraobserver variability in observers (**a**) B and (**b**) C. Differences are calculated as percentage of the mean volume, e.g., 100 × [B1 − B2]/([B1 + B2]/2). Dotted outer lines show the limits of 95% confidence intervals (mean ± 1.96s). Dot–dash lines show limits of 20% difference.

The mean overall intraobserver variability in both observers was +0.3% with a range of −39.6 to 51.5%. Separating the observers showed a mean difference in observer B of −2.3% (range: −39.6% to +51.5%) and in observer C of +2.8% (range: −29.4% to 34.3%). Bland–Altman plots (Figure 3) show a similar pattern of variation in both observers with a slight tendency of larger deviation in smaller testes. The proportion of measurements with a difference >20% was 18.6% (22/118 measurements). No differences were found in the two observers: 19% (11/58) in observer B and 18.3% (11/60) in observer C.

### 3.2. Interobserver Variability

**Figure 4:**
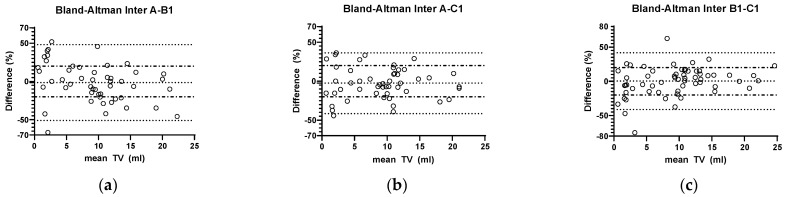
Bland–Altman diagram of interobserver variability in observer constellations (**a**) A-B1, (**b**) A-C1, and (**c**) B1-C1. Differences are calculated and data are shown in the same way as for intraobserver variability. No significant differences between the three observer conditions could be found (mixed effects model, *p* = 0.057). However, the scatter plots demonstrate a larger variability when compared to the intraobserver plots (cf. Figure 3).

The mean interobserver variability in all three available datasets (A-B1, A-C1, B1-C1, total of 160 measurements) was −1.0% (range: −74.1% to 62.8%). When splitting up between the three observers, no significant differences could be seen (Figure 4). Overall proportion of measurements with a difference >20% was 35% (56/160 measurements). When splitting up this proportion between the three interobserver settings, no significant changes could be seen (A-B1: 44.0% [22/50), A-C1: 34% [17/50], B1-C1: 28.3% [17/60], *p* = 0.22, chi-square test).

When comparing the overall rate of >20% difference between the intra- and interobserver groups, the differences were significant (*p* = 0.0027, chi-square test).

Next, in order to investigate which factors influence the accuracy of the TV measurements, various sub-analyses were carried out with the aim of determining the conditions that enable a sufficiently accurate TV determination.

### 3.3. Influence of Testicular Volume

**Figure 5:**
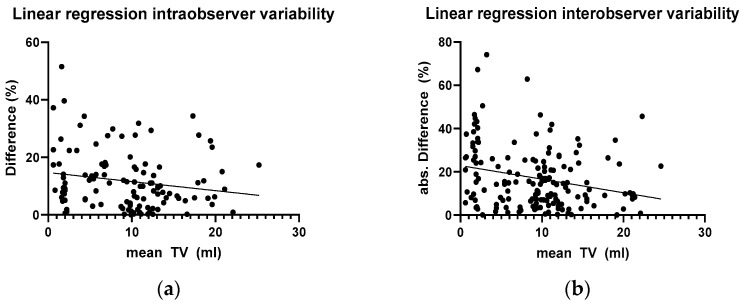
Linear regression analysis between mean TV and difference showed (**a**) a tendency towards a lower relative difference measurement in the intraobserver group (*p* = 0.054) and (**b**) a significantly lower difference in the interobserver group (*p* = 0.0012), albeit with a considerably higher spread.

To assess the influence of testicular volume, simple linear regression was performed both in the intraobserver and the interobserver group. In the interobserver group, a significant correlation could be found between a larger TV and a lower TV difference in %. In the intraobserver group, linear regression showed the same tendency, but not significant (Figure 5).

Next, in order to find out whether there is a sensible limit above which a testicular volume measurement has sufficient accuracy, a cut-off TV value of 4 mL was tested (chosen according to the graphic distribution in Figure 3 and Figure 4). In the intraobserver groups, 31.1% (9/28) of the measurements in testes below 4 mL TV showed a difference >20%, while this was only the case in 14.4% (13/90) of testes with a TV above this threshold, which was statistically significant (*p* = 0.035, chi-square test). This difference was even more pronounced in the interobserver group: in testes < 4 mL 63.2% (24/38) showed a >20% difference, while this was the case in larger testes only in 26.2% (32/122); *p* = 0.000031, chi-square test).

### 3.4. Influence of BMI

When comparing measurements in non-obese (BMI percentile < 90%) and obese (BMI percentile ≥ 90%) groups, the overall rate of >20% TV difference in the intraobserver group was significantly higher in the non-obese group, at 22.4% (17/76 measurements), when compared to the obese group, at 2.8% (1/36 measurements, *p* = 0.0084, chi-square test).

In the interobserver group, a total of 24% (12/50 available measurements) exceeded 20% TV difference in obese patients, while in the non-obese patients this rate was 40.8% (40/98). Here, too, the difference was statistically significant (*p* = 0.0427, chi-square test).

### 3.5. Consistency between Left and Right Testicle Measurements

Lastly, to assess the consistency in the difference between left and right testes, the degree of conformity regarding >20% difference (according to the EAU definition of a dystrophic testis) between the investigators in all patients was calculated. In total, 144 individual left-to-right difference measurements were available, 4 to 5 measurements per patient. In 44 of these (30.6%), a left-to-right difference > 20% was noted, which occurred in 23 of the 30 patients (76.7%). Only in seven patients (23.3%), no observer noted a >20% difference between both testes in any measurement. In 17 patients, only one or two of the observations showed this difference, while three or four observations of >20% left-to-right difference was only found in 3 patients each. No patient showed a consistent left-to-right difference > 20% in all available measurements. A Pearson’s r correlation matrix was calculated for the different investigators and showed a correlation range between 0.40 and 0.52 in the intraobserver group and between 0.05 and 0.69 in the interobserver group.

## 4. Discussion

In the assessment of testicular volume in pubertal boys, the Prader orchidometer has by and large been replaced by the use of ultrasound. It is important that the apparently very accurate measurement data that can be obtained using this method do not lead to a false sense of security, from which therapeutic decisions are then made. Recently, concerns about the validity of TV measurements in boys have been raised in several studies [10]. Notably, in the study by Welliver et al. a surprisingly high rate of intra- and interobserver variability was noted [11]. Our study aimed to investigate this still open question with a larger dataset with repeated measurements and different observers to possibly identify the conditions under which a reasonably accurate TV measurement is possible.

The age range of the probands included in this study was chosen to reflect the possible population for potential varicocele treatment; therefore, patients younger than 11 years were not included. Still, the large variety of testicular volume (0.5 mL to 27.4 mL) highlights the vast testicular growth during a considerably short period and the potential influence of this condition on the accuracy of the measurements. This is illustrated by the fact that a measurement of a 2 mL TV difference (as quoted in the EAU guidelines on pediatric urology) at a 10 mL level of TV—roughly the mean TV in our study—equals a 20% difference, while the same at the level of 20 mL equals a 10% difference.

While our results show a low mean overall intraobserver and interobserver variability (+0.3% and −1.0%, respectively) at the cohort level, the individual variability is quite concerning. In the intraobserver group, the rate of >20% variability was 18.6%, and 35% in the interobserver group.

This partly corroborates the findings of Oehme et al., who similarly found a low mean intraobserver and interobserver variability of 2.2% and 4.8%, respectively. They conclude, in concordance with our findings, that ultrasound-based TV measurement is suitable at the group level, such as population-based or epidemiological studies.

Based on their results, they concluded that intraobserver variability was low enough to justify longitudinal follow-up in individual boys. In our study, we could not reproduce this finding. While it was lower than in the study by Welliver et al. (18.6% compared to 25%), it was still considerably higher than in the study by Oehme et al. [10].

In contrast, interobserver variability was insufficient in all three studies, including our own. Notably, the rate of interobserver variability in our study matches the rate in the study by Welliver et al. (both 35%). Notably, in adults, interobserver variability has been shown to be low enough to justify ultrasound as a reproducible tool for longitudinal TV measurements [12]. However, the average testicular size in this study was approximately twice as high as in our patient collective and with an equally significantly smaller spread.

In order to assess the influence of testicular volume, we were able to show that above a TV of 4 mL, the accuracy in both the intra- and interobserver groups was significantly higher, albeit not to a level which allows unequivocal support of the method for longitudinal follow-up.

Of note, performing the examination by physicians with long-standing experience in ultrasound did show a moderately lower intraobserver variability but not a decrease in interobserver variability compared to the study by Welliver et al., where the examination was performed by ultrasound technicians [11]. This, too, indicates a fundamental limitation of the accuracy of the method and shows the limitations of longitudinal assessment of testicular volume.

Furthermore, an analysis of the left-to-right consistency showed only low-to-moderate consistency between different observers and a moderate consistency between different measurements of the same observers. Therefore, the rate of testes showing an asynchronous size in at least one of our measurements was also higher than that reported in the literature [2]. It must be assumed that at least part of this inconsistency has to be attributed to the limitations of the method, and therefore the validity of the ultrasound-based measurements of normal testicular growth synchronicity and volume difference must at least to a certain degree be questioned. Basing clinical decisions on a single measurement of a left-to-right difference of >20% seems at least to be doubtful.

In a recent review, an even more detailed approach using testicular asymmetry was proposed [16]. In their approach, varicocele repair should already be suggested in patients with a TV difference of >10–20% if at follow-up an adequate catch-up growth is not noted. Given the findings in our study, this alone seems too detailed and would require a much higher accuracy of testicular ultrasound volumetry. Therefore, additional diagnostic criteria should be used in decision making, such as peak retrograde flow (PRF) and sperm and/or hormonal abnormalities [16]. Potentially, elastosonography (a non-invasive technique to assess testicular elasticity), which has already been used in adult varicocele, may be of value in adolescent varicoceles as well [17].

Strengths and limitations: To the authors knowledge, this study represents the largest dataset available to assess the question of intra- and interobserver ultrasound validity in pubertal boys, with a larger set of replicate measures than previously available. This overcomes some of the limitations of earlier studies and renders our results potentially more accurate. However, there are also some limitations. Mean TV in our study (9.2 mL) was different from that of the previously published studies in (pre-)pubertal boys mentioned above (7.7 mL [10] and ~14 mL [11]), which makes comparison difficult due to volume effects, as discussed above. Although our intraobserver variability was lower than that in the study by Welliver et al. (with a higher mean TV), both our intraobserver and interobserver variability were higher than those in the study by Oehme et al. (with a lower mean TV). This finding as of now remains unexplained except for the fact that there are obviously significant differences even in mean TV between different investigators, even when the measurement method is standardized (cf. Figure 2), although this is not necessarily related to the length of clinical experience. Also, interobserver variability could not be calculated between different pediatric radiologists, which limits the applicability to clinical settings where testicular ultrasound is routinely performed by pediatric radiologists.

Furthermore, in our study the rate of obese patients was higher than expected at the population level. This may also lead to a bias, although interestingly we found a significantly lower rate of >20% TV difference in obese patients. The validity of this rather pronounced finding may be supported by the use of age-correlated BMI percentiles rather than the use of BMI alone as in previous studies [11].

This difference, one might speculate, is possibly due to the fact that in these patients the testes are supported upwards by the increased circumference of the upper leg, similar to a scrotal pad. Therefore, placing the testes in a preformed scrotal pad to support and lift the testicles in optimal position could potentially ensure a higher accuracy in TV measurements, especially in non-obese patients, similar to hip dysplasia ultrasound in newborns, where improper probe positioning substantially influencing the results can be rectified by use of a positioning aid [18]. On the other hand, supporting the scrotum did not lead to a sufficient accuracy in the study by Welliver et al. [11].

## 5. Conclusions

Both intraobserver and interobserver variability in ultrasound-based TV measurements in pubertal boys contain a relevant degree of uncertainty that renders this method unsuitable for individualized follow-up care, especially if therapeutic decisions are made solely dependent on fixed limit values. In particular, the use of absolute differences (in mL) should be avoided as a decisional tool due to the large differences in volume during pubertal growth. At the cohort level, however, mean differences in ultrasound-based TV measurements are low enough to make ultrasound comparisons reasonable.

## Figures and Tables

**Table 1:** Basic demographic data of the patients.

	Mean		Range
Age (yrs)	13.6		11.1–16.8
Testicular volume (all measurements) (mL)	9.2		0.5–27.4
BMI (kg/m^2^)	22.2		16.3–45.7
BMI percentile	60.9		4–99.8
	# of patients		%
Tanner stage (G)	G1	1	3.3%
	G2	7	23.3%
	G3	10	33.3%
	G4	7	23.3%
	G5	5	16.7%

## Data Availability

The data presented in this study are available on request from the corresponding author due to restrictions in Institutional Ethics Committee approval.
